# Venous Thromboembolism and Thymic Hyperplasia in the Setting of Silent Graves' Disease

**DOI:** 10.7759/cureus.23935

**Published:** 2022-04-07

**Authors:** Melanie N Rayan, Tyler S Jones, Ariel Ruiz de Villa, Matthew Calestino, Yvette Bazikian

**Affiliations:** 1 Internal Medicine, University of Central Florida College of Medicine/HCA Florida North Florida Hospital, Gainesville, USA

**Keywords:** deep venous thrombosis, thymic hyperplasia, thymic lymphoid hyperplasia, incidental thymic mass, clotting in thyroid disorders, dvt, venous thromboembolism, hypercoagulability in graves' disease

## Abstract

Venous thromboembolism is a common, yet serious life-threatening condition that has many well-recognized associations which include but are not limited to pregnancy, polycythemia, trauma, immobility, and malignancy. The pathophysiology behind the pro-coagulant effects of hyperthyroidism has been well established; however, there are no current guidelines regarding deep venous thrombosis (DVT) surveillance in patients with hyperthyroidism. In this report, we discuss the case of a 36-year-old female with no significant past medical history (PMH) with the exception of a 15 pack-year smoking history, who presented to us with an extensive, rapidly-progressing lower extremity DVT. Despite aggressive treatment measures, she developed a pulmonary embolus in the hospital. During her stay, she was diagnosed with Graves’ disease by hormone profile and thyroid-stimulating hormone receptor (TSH-R) antibody positivity. Additionally, an incidental thymic mass, likely thymic hyperplasia, was found on imaging and presumed to be associated with Graves’ disease. This case study reports a difficult-to-treat venous thromboembolism in the setting of Graves’ disease along with a review of current literature and pathophysiology on the subject.

## Introduction

Venous thromboembolism (VTE) is a multifactorial disease, with an incidence of one to two cases per 1,000 annually [1]. Several controlled and uncontrolled risk factors such as smoking, obesity, immobility, malignancy, and a family history of thrombophilic disorders can contribute to a hypercoagulable state, leading to VTE. 

In current literature, associations have been made between hyperthyroidism and a hypercoagulable state presenting in the form of thrombosis in the cerebral venous system [2]. However, no clear relationship between hyperthyroidism and venous thromboembolism has been established. In this case, we discuss the presentation of deep venous thrombosis (DVT) and pulmonary embolism (PE) in a young healthy patient with underlying asymptomatic Graves' disease. 

## Case presentation

**Figure 1:**
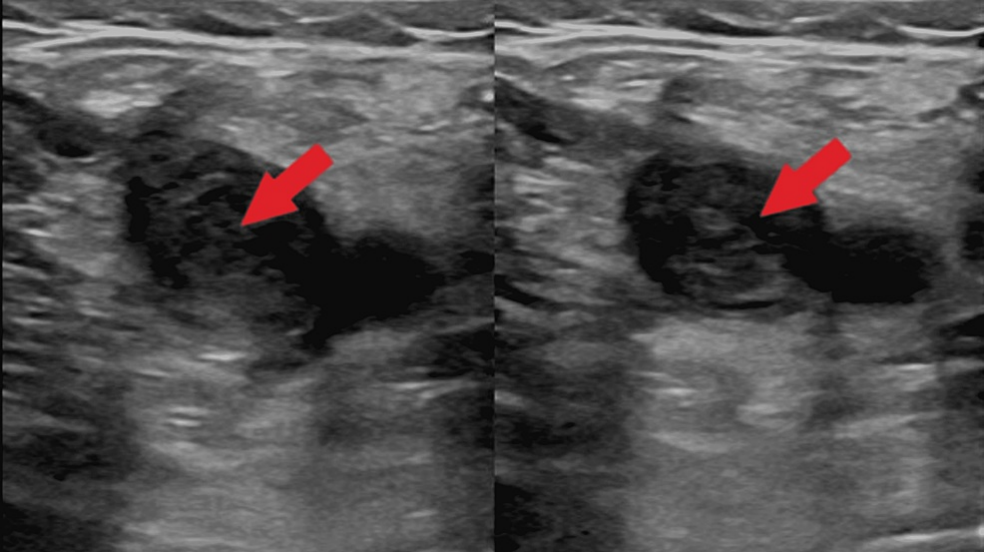
Non-compressible deep venous thrombosis (DVT) within the left common femoral vein

Our patient is a 36-year-old female with no known past medical history who presented with worsening pain in her left leg. A week prior to presentation, she was diagnosed with a DVT of the left external iliac vein extending to the distal femoral vein via ultrasonography (USG) during an urgent care visit and prescribed apixaban and oxycodone. Unrelenting symptoms of pain and swelling in her left lower extremity brought her to our ED. She complained of severe burning pain that was rated as 8/10 in severity and isolated to the left thigh. The pain was aggravated by ambulation and relieved by elevation and warm packs. A review of systems was notable for intermittent subjective fevers over the past month. A detailed history was obtained to determine if her DVT was provoked. She denied any recent travel or periods of immobility, trauma, surgery, history of spontaneous abortions, and any personal or family history of hypercoagulable disorders. She denied any medication use, including oral contraceptive pills. Social history was positive for a 15 pack-year smoking history, with continued ongoing smoking. Vital signs were found to be within the normal range, with no tachypnea or desaturations noted while breathing room air. Physical exam was significant for swelling, erythema, warmth, and tenderness of the medial and anterior aspects of the left thigh and knee, as well as numbness and decreased sensation in those territories. A significant difference in the diameter of the left and right thigh was also noted. Routine lab work was within normal limits, except for a D-Dimer of 2.91 mg/L (0.19-0.6 mg/L) and a fibrinogen level of 723 mg/dL (200-450 mg/dL). A urine pregnancy test was negative. Venous USG of the left leg showed a DVT, now extending to the popliteal vein, portrayed in Figure 1.

**Figure 2:**
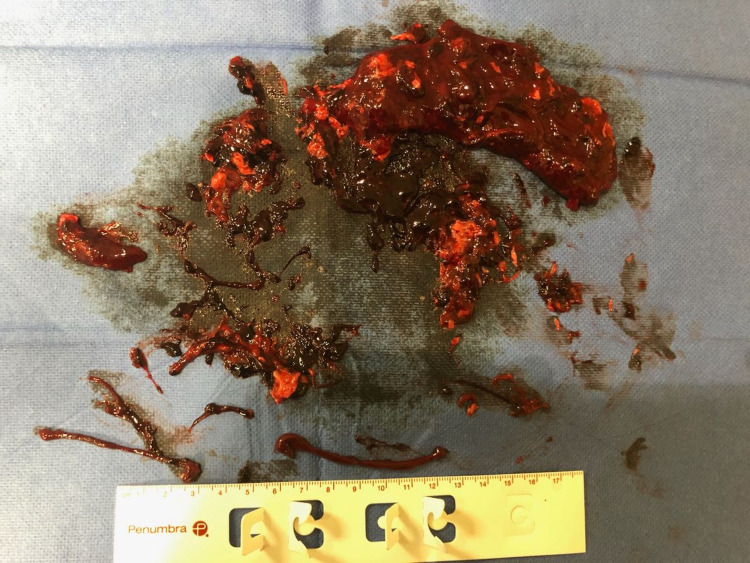
Gross finding of venous clot obtained from aspiration thrombectomy

**Figure 3:**
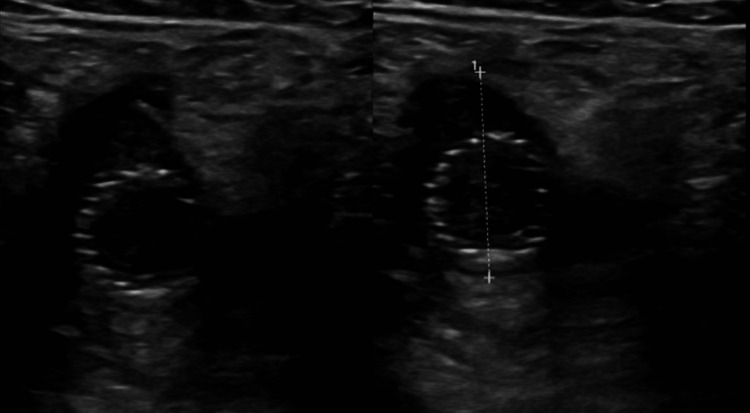
Thrombus formation within the stents

Interventional Radiology was consulted and an aspiration thrombectomy of the DVT was performed the same day with the administration of 5000 units of heparin and 6 mg of tissue plasminogen activator (TPA). The fibrinous thrombus resected can be visualized in Figure 2. Due to persistent occlusive flow-limiting lesions, stents were placed in the external iliac and common femoral veins. Follow-up venography demonstrated patency of the stents and the patient was subsequently started on a heparin drip. However, the following day, a repeat USG showed a recurring DVT, along with thrombosis within the stents as depicted in Figure 3. The heparin drip was continued, and the patient was promptly transferred to the ICU and treated with a combination of USG-guided catheter-directed pharmacologic thrombolysis and mechanical thrombectomies. No residual flow-limiting stenosis or thrombus was seen on the venogram following this procedure.

**Figure 4:**
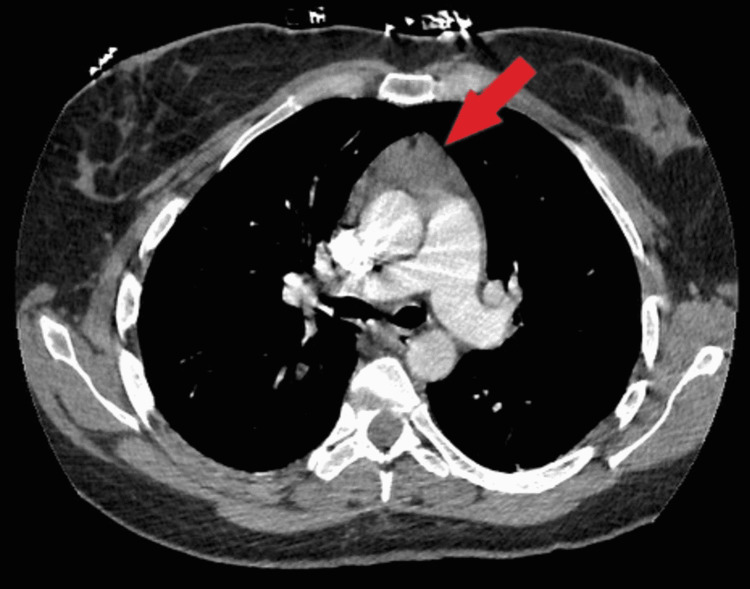
Incidental anterior mediastinal mass

On day five, the patient began to experience pleuritic chest pain and shortness of breath with tachycardia. Computed tomography angiography (CTA) demonstrated a large pulmonary embolus in a branch of the right lower lobe, along with an incidental soft tissue density measuring 2.4 x 4.4 mm visualized in Figure 4, concerning for a thymoma/ thymic mass. Given the need for multiple timed draws to measure Anti-Xa levels and activated partial thromboplastin time (aPTT) leading to difficulty in reaching adequate therapeutic goals on intravenous (IV) unfractionated heparin, she was switched to a weight-based twice-daily regimen of low molecular weight heparin (LMWH). Owing to the requirement for continued anticoagulation, a biopsy of the anterior mediastinal mass could not be performed during the patient's hospitalization. 

Clinical suspicion for thyroid disease prompted us to obtain a thyroid panel, which showed a hyperthyroid picture with undetectable thyroid-stimulating hormone (TSH), elevated thyroxine (T4) (18.3 mcg/dL), and free T4 (2.50 ng/dL) along with an elevated total tri-iodothyronine (T3) (217 mcg/dL) and free T3 (9.27 ng/dL) levels. Physical exam and thyroid ultrasound showed a normal-sized thyroid gland. Immunologic testing was positive for thyrotropin-receptor antibodies (TRAP) (17.50 IU/L), and thyroid-stimulating antibodies (TSI) (13.5 IU/L) consistent with the diagnosis of Graves’ disease. The patient was discharged on methimazole, atenolol, and LMWH, with instructions to follow up with Cardiothoracic Surgery for re-imaging and possible biopsy of the anterior mediastinal mass if deemed appropriate, once her thyroid indices normalized. Hypercoagulability work-up was initiated in the hospital with instructions to follow up for pending studies, including evaluation for possible malignancy.

## Discussion

In this case, we intend to demonstrate two primary points. The first is with respect to hyperthyroidism and the growing evidence that such a condition may induce a hypercoagulable state. High levels of thyroid hormone can have a potentiating effect on the coagulation system [3]. Hyperthyroidism favors a pro-coagulable and hypofibrinolytic state through its effects on Von Willebrand factor, Factors VIII, IV, X, fibrinogen, and plasminogen activator inhibitor-1 [4, 5]. Activated partial thromboplastin time is shortened and clot lysis time is prolonged as a result of changes in the levels of these factors. Factor VIII activity is markedly affected by tissue metabolic rate and plasma catecholamine levels, both phenomena that are significantly amplified in hyperthyroid patients.

Activation of thyroid hormone receptor beta (THRB) is one of the leading hypotheses with respect to exactly how thyroid hormone causes a hypercoagulable state [6,7]. Supporting this potential mechanism is the finding that patients with thyroid hormone resistance with defective THRB do not tend to develop increased coagulation factors, this being in spite of increased thyroid hormone. Factor VIII activity usually returns to normal after a few weeks of treatment with antithyroid therapy. Thus, correction of the hyperthyroid state is an important part of the treatment strategy [8]. These findings support the notion that physicians must be aware of the potential for DVTs in patients with hyperthyroidism. Whether lower extremity ultrasound on patients with elevated thyroid hormone in the setting of acute illness is indicated has yet to be determined. Regardless, we recommend adding hyperthyroidism to the list of differentials when evaluating a patient with an otherwise unprovoked clot.

A second matter we would like to drive home is the association between thymic hyperplasia and Graves’ disease. This association was first reported in the early 1900s [9]. As with our patient, thymic hyperplasia is often discovered as an incidental mediastinal mass on imaging. The exact mechanism of the hyperplastic changes is unclear but seems to involve a complex interaction of immunological and hormonal mechanisms [9]. Furthermore, thyroid-stimulating hormone receptors (TSH-R) are present within the thymus. These receptors are activated by anti-TSH-R antibodies. The resulting hyperplasia is similar to the tissue growth that results in Graves' orbitopathy, goiter, and pretibial myxedema. There are two subtypes of thymic hyperplasia [9]. First is true thymic hyperplasia, during which cortical and medullary parenchyma are hyperplastic. The second is thymic lymphoid hyperplasia, in which case lymphoid follicles are formed in excess [10]. Thymic lymphoid hyperplasia has been found to be the most common form of thymic hyperplasia in Graves' disease patients and is rarely cancerous. Treatment of Graves' disease has been shown to halt the growth of the thymus, often leading to resolution of the hyperplasia [10]. While an optimal course of treatment has not yet been established, we contend that medical treatment with periodic follow-up imaging is a plausible course of action. And because cancer is rarely found in such cases, surgical resection is typically not indicated. 

## Conclusions

The primary message that we would like to convey through this case report is for physicians to be mindful of underlying hyperthyroidism in all patients that present to the hospital with VTE, as early recognition and treatment is pivotal in preventing unfavorable outcomes in this patient population. In addition, we would like to exemplify the association between thymic hyperplasia and Graves’ disease, and emphasize its prompt resolution with medical management, eliminating the need for surgical resection.
